# High-Quality Draft Whole-Genome Sequences of 162 *Salmonella enterica* subsp. *enterica* Serovar Enteritidis Strains Isolated from Diverse Sources in Canada

**DOI:** 10.1128/genomeA.00348-14

**Published:** 2014-05-01

**Authors:** Muhammad A. Rehman, Kim Ziebell, John H. E. Nash, Andrew M. Kropinski, Zusheng Zong, Emily Nafziger, Patrick Boerlin, Linda Chui, John Devenish, Sadjia Bekal, Morag Graham, Roger P. Johnson

**Affiliations:** a Laboratory for Foodborne Zoonoses, Public Health Agency of Canada, Guelph, Ontario, Canada; b Department of Pathobiology, Ontario Veterinary College, University of Guelph, Guelph, Ontario, Canada; c Alberta Provincial Laboratory for Public Health, Edmonton, Alberta, Canada; d Department of Laboratory Medicine and Pathology, University of Alberta, Edmonton, Alberta, Canada; e Animal Health Microbiology Laboratory, Canadian Food Inspection Agency, Ottawa, Ontario, Canada; f Laboratoire de Santé Publique du Québec, Sainte-Anne-de-Bellevue, Quebec, Canada; g National Microbiology Laboratory, Public Health Agency of Canada, Winnipeg, Manitoba, Canada; h Department of Medical Microbiology, University of Manitoba, Winnipeg, Manitoba, Canada

## Abstract

We report the high-quality draft genome sequences of 162 strains of *Salmonella enterica* subsp. *enterica* serovar Enteritidis representing diverse phage types and pulsed-field gel electrophoresis (PFGE) profiles. The analysis of these genomes will enable the identification of markers that are useful for differentiating strains of this highly clonal serovar and will provide insights into the evolution, virulence, and epidemiology of the strains.

## GENOME ANNOUNCEMENT

*Salmonella* infections represent a major food-borne threat, with an estimated 93.8 million cases of gastroenteritis occurring globally each year, resulting in 155,000 deaths (1). In Canada, *Salmonella enterica* subsp. *enterica* serovar Enteritidis is among the three most common *Salmonella* serovars causing food-borne illness, most often associated with contaminated eggs and poultry. Over the past decade, the reported incidence of *S*. Enteritidis infections has more than doubled (2, 3), and it accounted for 40.6% of all reported cases in 2011 (4).

Because *S*. Enteritidis is genetically highly homogenous, standard pulsed-field gel electrophoresis (PFGE) and phage typing are of limited value as subtyping tools for outbreak detection and source attribution. Whole-genome sequencing and analysis overcome this limitation by providing the discriminatory power needed for differentiating such highly clonal strains (5), provided that genomes from a broad and diverse range of strains are available. Hence, we report here the availability of high-quality draft genome sequences of 162 *S*. Enteritidis strains from clinical (*n* = 3), environmental (*n* = 26), animal (*n* = 54), and food (*n* = 79) sources from diverse Canadian locations and belonging to 24 different phage types.

Genomic DNA was prepared by lysozyme-based cell lysis and subsequent extraction using the Qiagen EZ1 DNA tissue kit. The genomes of 13 *S*. Enteritidis strains were sequenced on two platforms: Roche 454 GS-FLX Titanium (at McGill University and Genome Québec, Montréal, Canada), achieving >40× average genome coverage, and the Illumina HiSeq 2500 (at the Centre for Applied Genomics, Hospital for Sick Children, Toronto, Canada), using the Illumina TruSeq DNA sample preparation kit with 2 × 101 paired-end runs, achieving >90× average genome coverage. The remaining 149 genomes were sequenced on the Illumina MiSeq platform (at the Public Health Agency of Canada [PHAC] National Microbiology Laboratory, Winnipeg, Canada) with 2 × 251 paired-end runs after library preparation with the Illumina Nextera XT sample preparation kit. The reads were analyzed and quality checked using FastQC (http://www.bioinformatics.babraham.ac.uk/projects/fastqc/). The combined Roche and Illumina HiSeq sequences of the first 13 genomes were generated by using the MIRA assembler version 4.0 (6) and by manually checking potential joins using the Gap5 software of the Staden package (7). The sequences of the remaining 149 genomes were assembled by aligning the contigs to the closely related genome of *S*. Enteritidis strain P125109 (GenBank accession no. AM933172) (8) using the MOSAIK software package (http://code.google.com/p/mosaik-aligner). The draft genomes consist of several contigs totaling from ~4,684,342 to 4,753,867 bp, with an average G+C content of 52.17%. The genomes were annotated with the National Center for Biotechnology Information (NCBI) Prokaryotic Genome Annotation Pipeline (PGAP) (http://ncbi.nlm.nih.gov/genomes/static/Pipeline.html), identifying on average a total of ~4,500 coding DNA sequences. No attempt was made to identify plasmid sequences in the draft genomes.

These 162 genome sequences have been deposited in GenBank, contributing to the increasing number and diversity of *S*. Enteritidis genomes available for analysis. Such analyses will provide unique insights and a better understanding of the virulence mechanisms and evolutionary history of this pathogen, as well as opportunities to develop more discriminatory subtyping tools. Further information and analyses of these isolates will be included in a forthcoming publication.

### Nucleotide sequence accession numbers.

The complete genome sequences of these 162 *S*. Enteritidis strains have been deposited in GenBank under Bioproject no. 219482. The GenBank accession no. are listed in Table 1.

**TABLE 1:** Accession and isolate numbers and sequencing methods for 162 *Salmonella* Enteritidis strains sequenced in this study

GenBank accession no.	Isolate accession no.	Original isolate no.	Sequencing method(s)^*a*^
CP007266	EC20110223	NML 5-6733	1, 2
CP007269	EC20121175	10-784	3
CP007270	EC20121176	CNG-006s.1	3
CP007271	EC20121178	H96EGG.1852MC	3
CP007272	EC20121179	H98MEAT.0020	3
CP007273	EC20121180	H96EGG.1622	3
CP007274	SA20093266	SA01CE09024801	3
CP007277	SA19930684	1243	3
CP007278	SA19942384	905X3	3
CP007279	SA19943269	1344X9-2	3
CP007280	SA19960848	203X6	3
CP007281	SA19961622	96-3071	3
CP007282	SA19970510	H96EGG.1852BS	3
CP007283	SA19970769	H97EGG.0001BUS	3
CP007284	SA19971331	96-3071	3
CP007285	SA19980677	H97EGG.1347	3
CP007286	SA19981522	H98MEAT.0028	3
CP007288	SA19981857	H98MEAT.0142	3
CP007289	SA19982831	H98EGG.0281	3
CP007290	SA19983126	5316E(R765)	3
CP007291	SA19992322	FH-1062	3
CP007292	SA19994216	465-3(T-RAM)	3
CP007293	SA20083456	08-029276	3
CP007294	SA20083636	08-0336268	3
CP007295	SA20084384	SA02DT08097701	3
CP007296	SA20090419	SA01AB08108401	3
CP007297	SA20090435	SA01AB08114401	3
CP007298	EC20090530	CE-M-09-4058	3
CP007300	SA20090877	SA02DT09011701	3
CP007301	SA20091739	SA02DT09053901	3
CP007302	SA20093421	SA01AB09056501	3
CP007303	SA20093430	SA01AB09058001	3
CP007304	SA20093538	SA02DT09081001	3
CP007305	SA20093543	SA02DT09083201	3
CP007306	SA20093784	SA02DT09101801	3
CP007307	SA20093788	SA02DT09104001	3
CP007308	SA20093950	SA02DT09115301	3
CP007309	SA20093977	SA01AB09073701	3
CP007310	SA20094079	SA02DT09134601	3
CP007311	SA20094350	09-077217-1A	3
CP007312	SA20094352	09-077411-1B	3
CP007313	SA20094383	SA02DT09036201	3
CP007314	SA20094389	SA02DT09149501	3
CP007315	SA20094521	SA02DT09171101	3
CP007316	SA20094642	SA01AB09100201	3
CP007317	SA20094803	H09-081233-6	3
CP007318	SA20095309	CE-W-09-0079	3
CP007319	SA20095440	09-095994-3A	3
CP007320	EC20090135	SA02DT08051801	1, 2
CP007321	EC20090193	SA02DT08109401	1, 2
CP007322	EC20090332	SA02DT08186901	1, 2
CP007323	EC20110222	NML5-6566	1, 2
CP007324	EC20111514	SA01AB10056401	3
CP007325	EC20111515	SA01AB10056601	3
CP007326	EC20111554	SA01AB10096801	3
CP007327	EC20111561	SA01AB10101301	1
CP007328	EC20111576	SA01AB10109701	1
CP007330	EC20120003	SE20050013	1, 2
CP007331	EC20120007	SA20110131	1, 2
CP007332	EC20120916	CE-M-12-4021	3
CP007333	EC20121177	CE-R2-12-0008	3
CP007334	SA20092320	SA01AB09038901	3
CP007335	EC20120580	SA01AB11007601	3
CP007336	EC20120581	SA01AB11007701	3
CP007337	EC20120590	SA01AB11021201	3
CP007338	EC20120597	SA01AB11031201	3
CP007339	EC20120685	SA01AB11073901	3
CP007340	EC20120686	SA01AB11074001	3
CP007341	EC20120687	SA01AB11074101	3
CP007342	EC20120697	SA01AB11085301	3
CP007343	EC20120722	CE-R2-12-0049	3
CP007344	EC20120213	SA02DT10090901	3
CP007345	EC20120219	SA02DT10101501	3
CP007346	EC20120229	SA02DT10107401	3
CP007347	EC20120240	SA02DT10132601	3
CP007348	EC20120356	CE-M-11-3112	3
CP007349	EC20120469	SA02DT10034701	3
CP007350	EC20120496	SA02DT10182801	3
CP007351	EC20120497	SA02DT10183701	3
CP007352	EC20120498	SA02DT10185301	3
CP007353	EC20120505	SA02DT10196301	3
CP007354	EC20120528	SA02RET100045201	3
CP007355	EC20100088	SA01AB09037401	3
CP007356	EC20100089	SA01AB09037701	3
CP007357	EC20100100	SA02DT09081001	3
CP007358	EC20100130	SA01AB09080501	1, 2
CP007359	EC20100134	SA01AB09081701	1, 2
CP007360	EC20100325	CE-V-10-0114	3
CP007361	SA20100349	10-604	3
CP007362	EC20120544	SA02DT10216401	3
CP007363	EC20120548	SA02DT10225701	3
CP007364	EC20120555	SA02DT10232501	3
CP007365	EC20120994	CE-R2-12-0101	3
CP007366	EC20121004	CE-R2-12-1021	3
CP007367	EC20121541	CE-R2-12-1032	3
CP007368	EC20121542	CE-R2-12-1033	3
CP007369	EC20121671	CE-R2-12-0164	3
CP007370	EC20121672	CE-R2-12-0165	3
CP007371	EC20121689	CE2-R2-12-2031	3
CP007372	SA20121703	CE-R2-12-0049	3
CP007373	EC20121744	SA02DT11110901	3
CP007374	EC20121746	SA02DT11111701	3
CP007375	EC20120925	CE-M-12-2028	3
CP007376	EC20120927	CE-M-12-4036	3
CP007377	EC20120963	CE-R2-12-0115	3
CP007378	EC20120968	CE-R2-12-0125	3
CP007379	EC20120969	CE-R2-12-0126	3
CP007380	EC20120970	CE2-R2-12-1026	3
CP007381	EC20121812	SA20114101	3
CP007382	EC20121825	SA20116251	3
CP007383	EC20121826	SA20116252	3
CP007384	EC20121969	CE-R2-12-0197	3
CP007385	EC20121970	CE-R2-12-0199	3
CP007386	EC20121976	CE-R2-12-0208	3
CP007387	EC20121986	CE2-R2-12-1047	3
CP007388	EC20121989	CE-R2-12-2044	3
CP007395	EC20121748	SA02DT11126101	3
CP007396	EC20121750	SA02DT11133401	3
CP007397	EC20121751	SA02DT11145101	3
CP007398	EC20121753	SA02DT11180801	3
CP007400	EC20120734	CE-R2-12-3015	3
CP007401	EC20120738	CE-R2-12-3020	3
CP007402	EC20120765	SA01AB11116101	3
CP007403	EC20120773	CE-R-12-2021	3
CP007404	EC20120774	CE-R-12-2023	3
CP007405	EC20120775	CE-R-12-3043	3
CP007406	EC20120776	CE-R2-12-0055	3
CP007407	EC20120917	CE-M-12-4022	3
CP007408	EC20120918	CE-M-12-4023	3
CP007411	EC20121990	CE-R2-12-2045	3
CP007412	EC20122022	CE-R-12-2073	3
CP007413	EC20122026	CE-R2-12-0230	3
CP007414	EC20122031	CE-R2-12-0241	3
CP007415	EC20122033	CE-R2-12-0246	3
CP007416	EC20122045	CE-R2-12-3088	3
CP007417	SA20123395	S31824-1.1	3
CP007418	EC20130345	SA01AB08065301	3
CP007419	EC20130346	SA01AB10104701	3
CP007420	EC20100103	SA02DT09083201	1, 2
CP007421	EC20090884	CE-W-09-0079	1, 2
CP007422	EC20090531	CE-M-09-4059	1, 2
CP007423	EC20130347	SA01AB10101001	3
CP007424	EC20130348	SA01AB10101201	3
CP007425	SA20082034	08-0183053	3
CP007426	SA20085285	08-0351346	3
CP007427	SA20100239	10-001197-5A	3
CP007428	EC20120677	SA01AB11059601	3
CP007429	EC20121765	SA02DT11281901	3
CP007430	EC20090195	SA02DT08110601	3
CP007431	SA20094682	09-15458 A	3
CP007432	EC20100131	SA01AB09080601	3
CP007433	EC20120051	CE-M-11-3106	3
CP007434	EC20120200	SA02RET100003701	3
CP007438	EC20120009	SE20080075	1, 2
CP007463	EC20120929	CE-M-12-4038	3
CP007464	EC20121747	SA02DT11112801	3
CP007465	SA19940857	LW175	3
CP007466	SA20084644	08-042758-1A	3
CP007467	SA20084824	H08-041885-26A	3
CP007468	SA20094177	CE-R-09-0262	3
CP007469	SA20094301	09.1424.7	3
CP007498	EC20111510	SA01AB10051101	3

a 1 = Roche 454, 2 = Illumina HiSeq, and 3 = Illumina MiSeq.

## References

[1] Majowicz SE Musto J Scallan E Angulo FJ Kirk M O’Brien SJ Jones TF Fazil A Hoekstra RM International Collaboration on Enteric Disease ‘Burden of Illness’ Studies 2010 The global burden of nontyphoidal *Salmonella* gastroenteritis. Clin. Infect. Dis. 50:882–889. 10.1086/650733 20158401

[2] Nesbitt A Ravel A Murray R McCormick R Savelli C Finley R Parmley J Agunos A Majowicz SE Gilmour M Canadian Integrated Program for Antimicrobial Resistance Surveillance Public Health Partnership, Canadian Public Health Laboratory Network 2012 Integrated surveillance and potential sources of *Salmonella* Enteritidis in human cases in Canada from 2003–2009. Epidemiol. Infect. 140:1757–1772. 10.1017/S0950268811002548 22166269 PMC3443964

[3] Landry L Dutil L 2010 Epidemiology of SE in humans in Canada. Canadian Salmonella Enteritidis Control Symposium, Vancouver, British Columbia, Canada, 1 December 2010

[4] Public Health Agency of Canada (PHAC). 2011 National enteric surveillance program (NSEP) annual summary. Public Health Agency of Canada, Winnipeg, Manitoba, Canada

[5] Zheng J Keys CE Zhao S Meng J Brown EW 2007 Enhanced subtyping scheme for *Salmonella* Enteritidis. Emerg. Infect. Dis. 13:1932–1935. 10.3201/eid1312.070185 18258051 PMC2876743

[6] Chevreux B Wetter T Suhai S 1999 Genome sequence assembly using trace signals and additional sequence information, p 45–56 *In* Computer science and biology. Proceedings of the German Conference on Bioinformatics, GCB ’99 GCB, Hannover, Germany

[7] Staden R Beal KF Bonfield JK 2000 The Staden package, 1998. Methods Mol. Biol. 132:115–130. 10.1385/1-59259-192-2:115 10547834

[8] Thomson NR Clayton DJ Windhorst D Vernikos G Davidson S Churcher C Quail MA Stevens M Jones MA Watson M Barron A Layton A Pickard D Kingsley RA Bignell A Clark L Harris B Ormond D Abdellah Z Brooks K Cherevach I Chillingworth T Woodward J Norberczak H Lord A Arrowsmith C Jagels K Moule S Mungall K Sanders M Whitehead S Chabalgoity JA Maskell D Humphrey T Roberts M Barrow PA Dougan G Parkhill J 2008 Comparative genome analysis of *Salmonella* Enteritidis PT4 and *Salmonella gallinarum* 287/91 provides insights into evolutionary and host adaptation pathways. Genome Res. 18:1624–1637. 10.1101/gr.077404.108 18583645 PMC2556274

