# Correction for Rehman et al., High-Quality Draft Whole-Genome Sequences of 162 *Salmonella enterica* subsp. *enterica* Serovar Enteritidis Strains Isolated from Diverse Sources in Canada

**DOI:** 10.1128/genomeA.01008-14

**Published:** 2014-09-18

**Authors:** Muhammad A. Rehman, Kim Ziebell, John H. E. Nash, Andrew M. Kropinski, Zusheng Zong, Emily Nafziger, Patrick Boerlin, Linda Chui, John Devenish, Sadjia Bekal, Morag Graham, Roger P. Johnson

**Affiliations:** a Laboratory for Foodborne Zoonoses, Public Health Agency of Canada, Guelph, Ontario, Canada; b Department of Pathobiology, Ontario Veterinary College, University of Guelph, Guelph, Ontario, Canada; c Alberta Provincial Laboratory for Public Health, Edmonton, Alberta, Canada; d Department of Laboratory Medicine and Pathology, University of Alberta, Edmonton, Alberta, Canada; e Animal Health Microbiology Laboratory, Canadian Food Inspection Agency, Ottawa, Ontario, Canada; f Laboratoire de Santé Publique du Québec, Sainte-Anne-de-Bellevue, Quebec, Canada; g National Microbiology Laboratory, Public Health Agency of Canada, Winnipeg, Manitoba, Canada; h Department of Medical Microbiology, University of Manitoba, Winnipeg, Manitoba, Canada

## AUTHOR CORRECTION

Volume 2, no. 2, e00348-14, 2014. Page 3: The first paragraph of the Acknowledgments should read as follows. “We sincerely thank the NCBI PGAP team for genome annotation services and Shaun Tyler at the PHAC National Microbiology Laboratory and staff at McGill University, Genome Québec, and the Centre for Applied Genomics, Toronto Hospital for Sick Children, for performing the sequencing. We also acknowledge provision of strains and related epi-data by Jane Parmley and Agnes Agunos, PHAC Canadian Integrated Program for Antimicrobial Resistance Surveillance (CIPARS); Frank Pollari and Katarina Pintar, FoodNet Canada; Neil Pople, Manitoba Agriculture, Food and Rural Initiatives; Hugh Whitney, Animal Health Laboratory, Department of Natural Resources, Newfoundland & Labrador; Durda Slavic and Carlos Leon-Velarde, Laboratory Services Division, University of Guelph; Valerie Bohaychuk, Alberta Agriculture and Rural Development; and Jane Pritchard, BC Ministry of Agriculture, British Columbia.”

